# Occult renal involvement by sigmoid colon adenocarcinoma presenting as spontaneous renal rupture: a case report

**DOI:** 10.3389/fsurg.2026.1954420

**Published:** 2026-10-30

**Authors:** Jun Yin, Chi Huang, Xiaohui Wei, Nan Zhang, Zhaoyu Xing, Li Cui

**Affiliations:** 1 The Department of Urology, Changzhou Maternal and Child Health Care Hospital, Changzhou Medical Center, Nanjing Medical University, Changzhou, China; 2 The Department of Urology, The Third Affiliated Hospital, Soochow University, Changzhou, Jiangsu, China

**Keywords:** case report, colorectal cancer, occult renal involvement, spontaneous renal rupture, upper urinary tract obstruction

## Abstract

Spontaneous renal rupture (SRR) is a rare nontraumatic emergency, usually associated with renal neoplasms, vascular lesions, infection, coagulopathy, or obstruction. Diffuse malignant infiltration may be missed when it does not form a discrete mass. A 45-year-old man previously treated for sigmoid colon adenocarcinoma had chronic left ureteral obstruction and presented with sudden severe left flank pain and gross hematuria. Contrast-enhanced CT showed left renal rupture with a large perirenal and retroperitoneal hematoma, a chronic left ureteral stent, and moderate right hydroureteronephrosis. No enhancing renal or ureteral mass was visible. Hemoglobin was 108 g/L on arrival, and the extent of the hematoma raised concern for ongoing hemorrhage. Selective renal arterial embolization was considered, but open nephrectomy was chosen because the left kidney was chronically compromised and tumor-related rupture could not be excluded. The right ureter was stented first to protect the remaining renal unit. Gross examination identified a dorsal cortical tear but no discrete tumor. Histology disclosed diffuse poorly differentiated carcinoma with extensive necrosis and invasion of renal hilar vessel walls. Immunohistochemistry supported colorectal origin. Urinary tract and systemic recurrence followed, and the patient died of multiorgan failure in June 2024.Diffuse malignant renal involvement should remain in the differential diagnosis when a patient with previous colorectal cancer develops unexplained nontraumatic renal or perirenal hemorrhage, even if imaging shows no mass. If nephrectomy is required in the presence of contralateral upper urinary tract obstruction, drainage of the remaining renal unit should be planned beforehand.

## 1 Introduction

Spontaneous renal rupture (SRR) is a rare nontraumatic urological emergency characterized by bleeding into the perirenal or retroperitoneal space. It should be distinguished from rupture of a renal fornix or the renal pelvis, where urinary extravasation predominates (1–3). Causes include renal neoplasms, vascular disorders, infection, coagulopathy, and obstructive uropathy. Contrast-enhanced CT is usually the first test used to identify the source (4–6).

Diagnosis becomes difficult when hemorrhage distorts the renal contour and CT shows no causative mass. Renal involvement by colorectal cancer (CRC) is uncommon and usually appears as a discrete renal or upper urinary tract lesion (7–14). Wolff et al. reported spontaneous rupture of a renal metastasis from colon adenocarcinoma in 1994 (13). In our patient, CT, operative inspection, and gross examination all failed to identify a mass, although microscopy showed diffuse tumor infiltration and invasion of hilar vessel walls. Obstruction of the opposite upper urinary tract also influenced emergency treatment.

After colorectal origin has been established, the route of renal involvement still be uncertain. Possible pathways include hematogenous or lymphatic dissemination, retrograde spread along the urinary tract, and direct extension through the retroperitoneum. Immunohistochemistry can distinguish colorectal carcinoma from primary renal and urothelial malignancies, but it cannot determine how the tumor reached the kidney.

We report spontaneous renal parenchymal rupture with perirenal and retroperitoneal hemorrhage in a man previously treated for sigmoid colon adenocarcinoma. Imaging, operative inspection, and gross examination showed no mass, whereas histology revealed diffuse renal involvement. Because the opposite upper urinary tract was also obstructed, its drainage had to be considered before nephrectomy. The report follows the CARE guidelines and SCARE 2023 criteria (15, 16).

## 2 Case presentation

### 2.1 Initial primary malignancy and baseline urinary status

In August 2019, a 42-year-old man was admitted to Fudan University Shanghai Cancer Center for elective treatment of biopsy-proven sigmoid colon adenocarcinoma. Preoperative imaging showed left hydronephrosis and close contact between the sigmoid lesion and the left ureter (Figure 1). Preoperative PET/CT showed increased FDG uptake in the sigmoid lesion, which was inseparable from the adjacent left distal ureter (Figure 2). Cystoscopy on August 9 showed smooth bladder mucosa without intravesical tumor, and a left double-J (D-J) stent was placed. Radical sigmoid colectomy was performed on August 12. The infiltrative tumor measured about 5 × 6 cm, penetrated the serosa, and partly encased the left ureter and adjacent iliac vessels. The operative report described dissection of the tumor from these structures without ureteral or vascular resection. No gross liver or peritoneal metastases were found, and all resection margins were negative.

**Figure 1:**
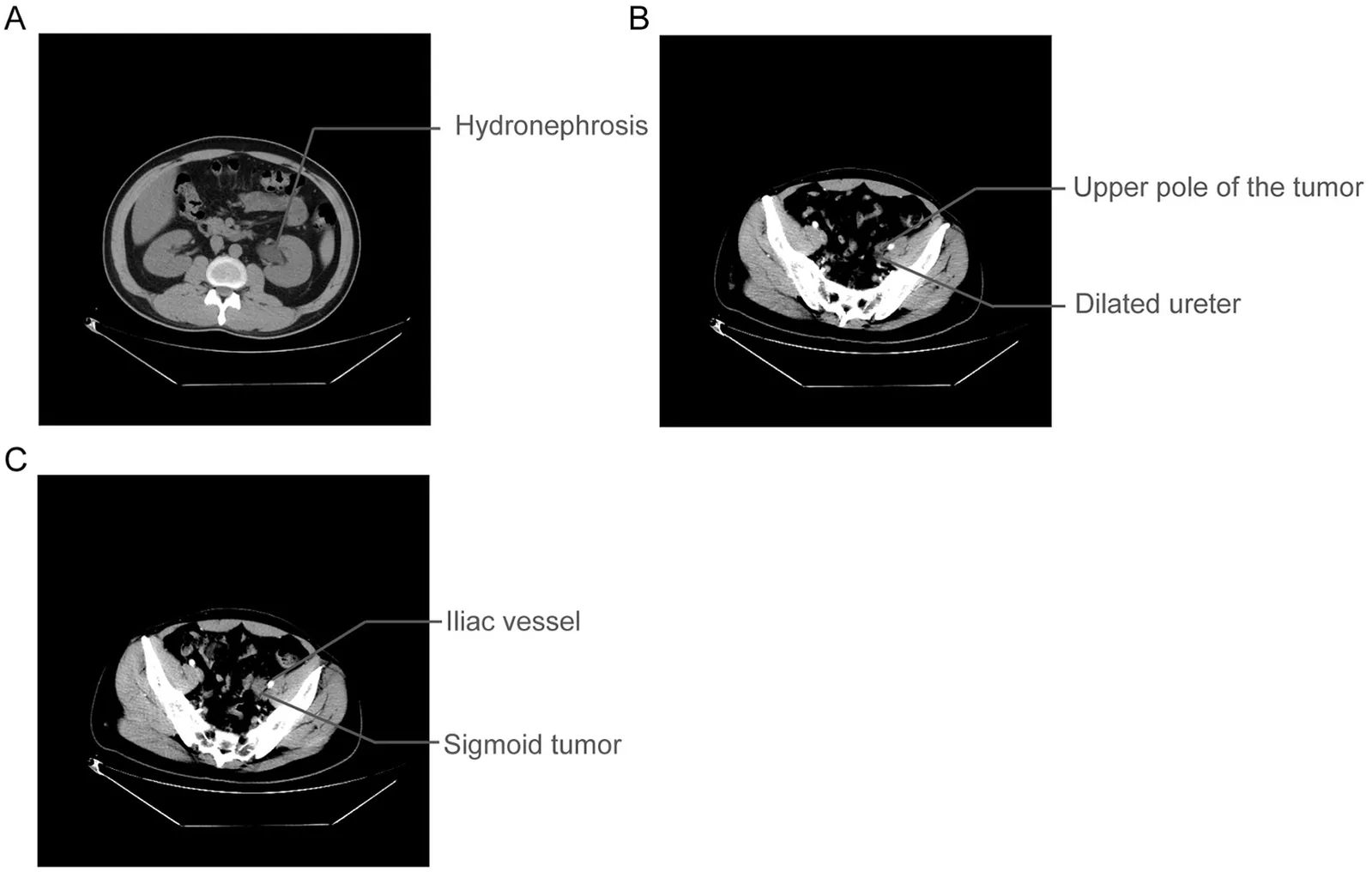
Primary sigmoid tumor with left upper urinary tract obstruction. **(A)** CT showed left hydronephrosis without a discrete left renal mass or obvious right hydronephrosis. **(B,C)** Contrast-enhanced CT showed the sigmoid tumor abutting or encasing the left ureter and adjacent iliac vessels, with dilatation of the proximal left ureter.

**Figure 2:**
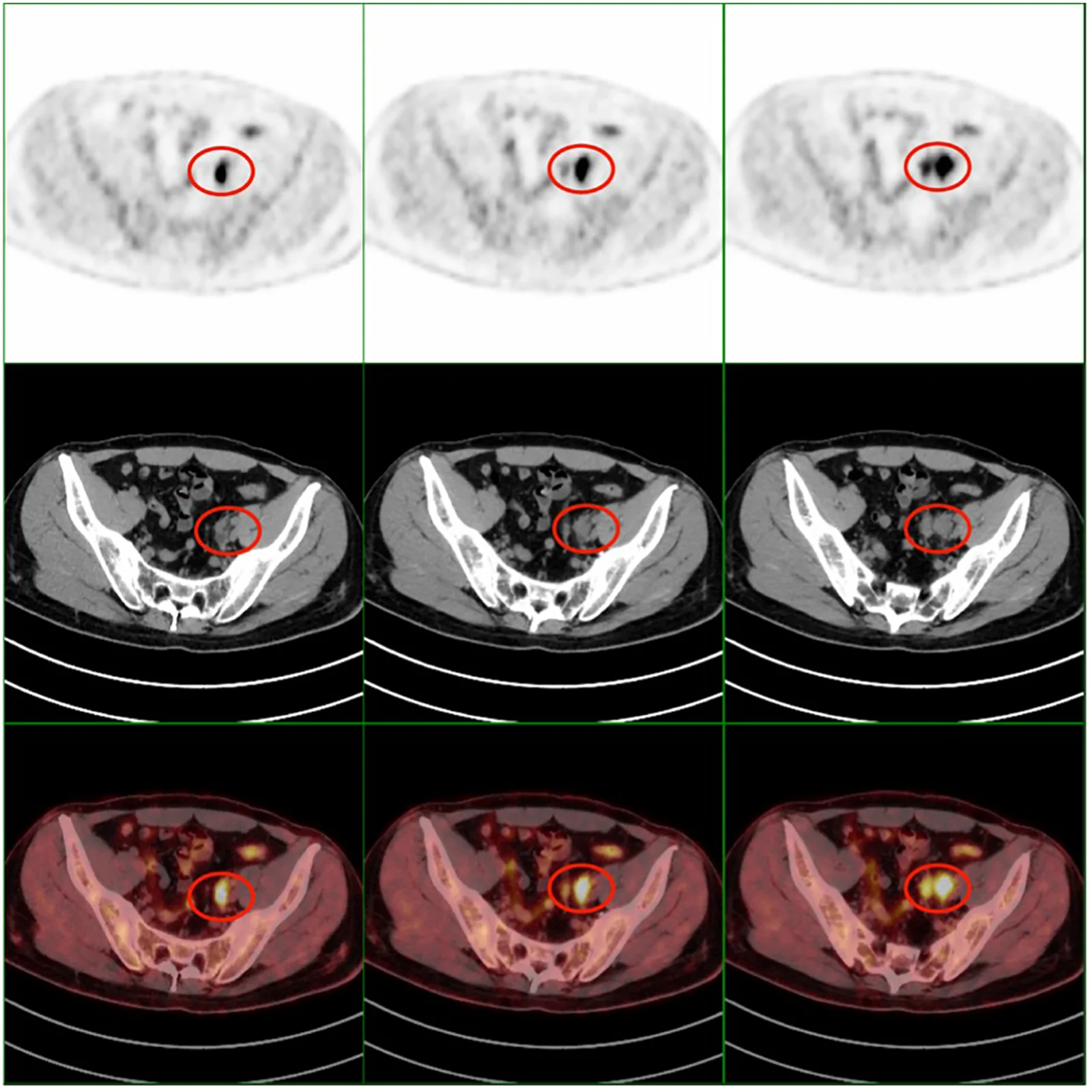
Preoperative PET/CT of the primary sigmoid lesion. PET/CT showed increased FDG uptake in the sigmoid lesion, which was inseparable from the adjacent left distal ureter on the available images.

Histopathology showed moderately to poorly differentiated adenocarcinoma, with vascular tumor thrombi and perineural invasion. Metastases were identified in 2 of 7 mesenteric nodes and 1 of 3 nodes labeled ‘highest lymph nodes’ in the original report. The reported stage was pT4bN1bM1a, although the lesion underlying the M1a designation was not specified. Metastatic disease may therefore have been suspected in 2019, but the record does not show that the kidney was involved at that time. The tumor retained MLH1, MSH2, MSH6, and PMS2; KRAS, NRAS, and BRAF were wild type, and the tumor was microsatellite stable. SATB2 and CDX2 expression supported colorectal differentiation. Left upper urinary tract obstruction persisted after colectomy and required D-J stent exchange about every 3 months. An oncology summary documented eight cycles of capecitabine plus oxaliplatin (XELOX), with the final cycle on February 7, 2020, followed by surveillance. CT on January 27, 2021, showed persistent dilatation of the left renal pelvis and ureter, and another left D-J stent was placed on March 17. The records did not identify a definite cause for the continuing obstruction.

### 2.2 Acute urological emergency and workup

On January 11, 2022, approximately 29 months after colectomy, the patient presented to our emergency department with 5 h of sudden severe left flank pain and gross hematuria. He reported no recent trauma, strenuous activity, or invasive urological procedure. Blood pressure was 132/91 mmHg, heart rate 110 beats/min, respiratory rate 18 breaths/min, temperature 35.7 °C, and oxygen saturation 98% on room air. Examination showed marked left costovertebral-angle tenderness without a palpable abdominal mass or peritonism. Blood tests obtained that day showed leukocytosis (16.91 × 10^9/L), hemoglobin of 108 g/L, and a platelet count of 206 × 10^9/L. Serum creatinine was 329 μmol/L (reference range, 46–96 μmol/L); no immediate pre-event value was available.

Emergency contrast-enhanced CT showed an irregular left renal contour and a large perirenal and retroperitoneal hematoma containing hyperattenuating blood products. The chronic left ureteral stent remained in place without obvious migration, although its patency could not be determined. Moderate right hydroureteronephrosis was also present. No discrete enhancing renal or ureteral mass, or focal lesion separate from the hematoma, was identified (Figure 3A). Left renal rupture was later confirmed at surgery and on gross examination. In the absence of trauma or a visible mass, possible causes included occult neoplasm, vascular or coagulation-related bleeding, infection, and obstruction-related rupture.

**Figure 3:**
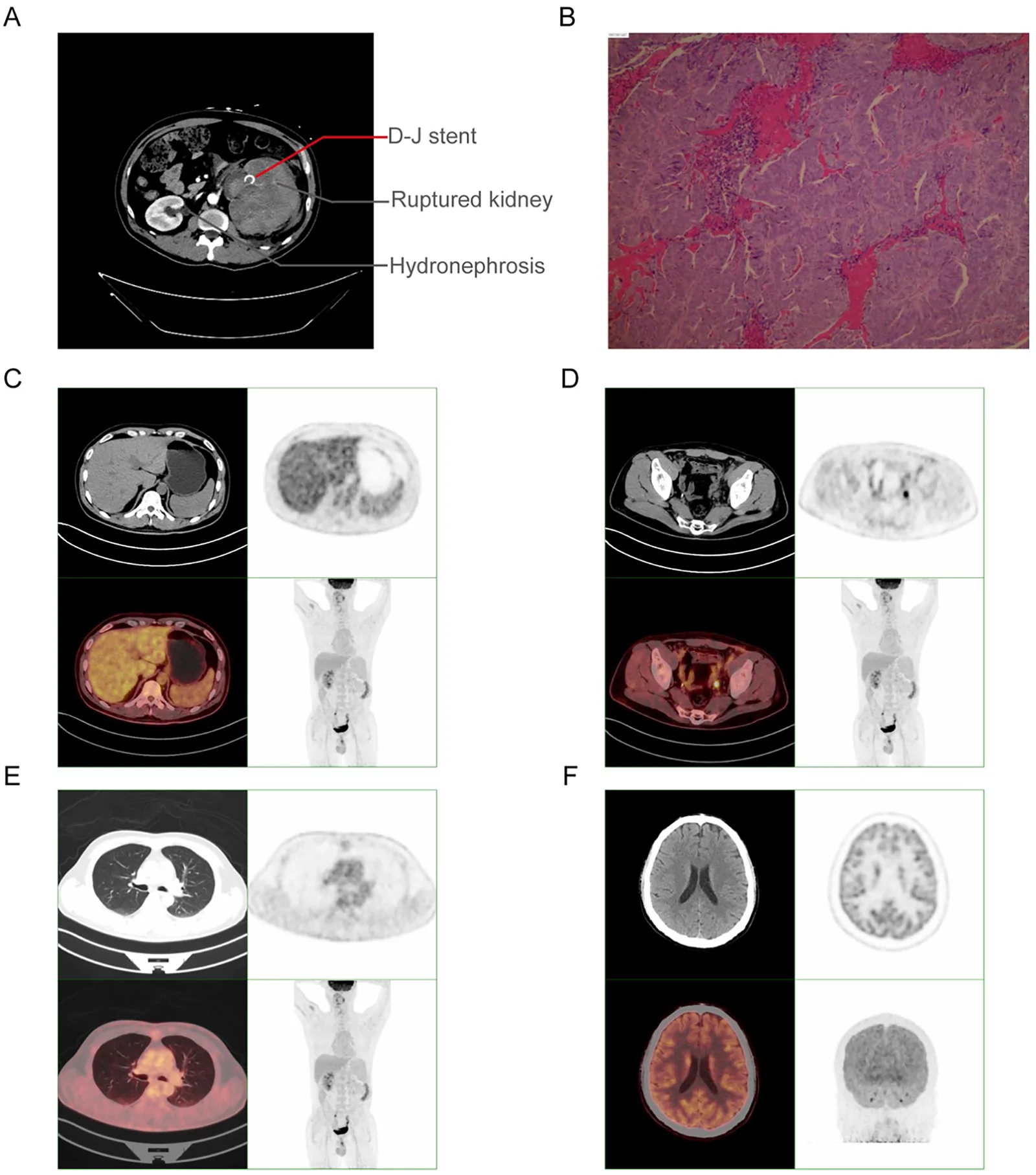
Emergency imaging, histopathology, and postoperative restaging. **(A)** Axial contrast-enhanced CT showed an irregular left renal contour with a large perirenal and retroperitoneal hematoma, the chronic left ureteral stent *in situ*, and right hydroureteronephrosis. No discrete enhancing renal mass was visible. **(B)** Hematoxylin and eosin staining of the nephrectomy specimen showed poorly differentiated carcinoma diffusely infiltrating renal tissue, with extensive necrosis and direct invasion of renal hilar vessel walls in the available section. Immunohistochemical findings are detailed in the text. **(C–F)** Postoperative PET/CT images are provided for descriptive context. Available records document studies on January 25 and August 5, 2022. Because the retained image export could not be assigned confidently to either examination, these panels were not used to infer isolated disease.

### 2.3 Surgical intervention and histopathological diagnosis

Management had to control the hemorrhage without compromising the right kidney. The large hematoma, anemia, and clinical course raised concern for continued bleeding. The left renal unit had long been obstructed and repeatedly stented and was considered chronically compromised; tumor-related rupture also remained possible. Selective renal arterial embolization was considered as a kidney-preserving option. After informed consent and preoperative review, open nephrectomy was chosen because bleeding appeared to be continuing, little function was expected to be salvageable in the obstructed kidney, and malignant rupture could not be excluded. Since the obstructed right kidney would become the only renal unit, a retrograde right ureteral D-J stent was inserted within about 2 h of admission. Open left nephrectomy followed approximately 3–4 h after admission. The cause of the right-sided obstruction was not known at that time.

At surgery, an extensive perirenal and retroperitoneal hematoma confirmed recent major hemorrhage. The pre-existing left ureteral stent was removed during *en bloc* nephrectomy. Gross examination showed a dorsal cortical laceration with surrounding hemorrhage but no discrete tumor. The operation lasted about 180 min. The operative note recorded an estimated blood loss of 400 mL and transfusion of 6 U of red blood cells and 600 mL of plasma. Hemoglobin was 94 g/L later that day and 83 g/L the next morning.

Microscopy disclosed poorly differentiated carcinoma permeating the renal tissue, with extensive geographic necrosis and direct invasion of renal hilar vessel walls (Figure 3B). Tumor cells expressed AE1/AE3, CK20, Villin, and CDX2, and the Ki-67 index was approximately 70%. CK7, PAX8, GATA3, and P40 were negative. The immunophenotype supported colorectal adenocarcinoma involving the kidney rather than a primary renal or urothelial malignancy. It did not identify an anatomical epicenter or distinguish among hematogenous, lymphatic, retrograde urinary tract, and contiguous retroperitoneal spread.

### 2.4 Postoperative course and oncological outcomes

Recovery was uncomplicated, with no complication documented within 30 days. PET/CT performed on January 25 showed uptake in the operative bed, left abdominal wall, and dilated left ureter; the report interpreted these findings as postoperative change. The case was reviewed by a multidisciplinary team on January 27, and the patient received four cycles of FOLFIRI between February and March 2022.

CT on March 28 showed persistent left hydroureteronephrosis, with thickening and enhancement of the distal left ureter and its intramural segment along the posterior bladder wall. A right ureteral D-J stent was placed ureteroscopically on April 1. Cetuximab plus FOLFOX began on April 7. On April 20, transurethral resection of the bladder lesion was performed together with right D-J stent exchange and right ureteral biopsy. The bladder specimen contained moderately differentiated adenocarcinoma consistent with colorectal origin; CK20, Villin, and CDX2 were positive, whereas CK7, GATA3, and P40 were negative. The right ureteral biopsy showed only mildly atypical urothelium and did not explain the obstruction. Disease remained stable after five cycles of cetuximab plus FOLFOX. Cetuximab was then stopped because of a rash, while FOLFOX continued through cycle 8 and ended on August 13. PET/CT on August 5 showed no abnormal FDG uptake at the colorectal anastomosis or bladder wall, but dense tissue in the left nephrectomy bed and a small right iliac perivascular lymph node were FDG-avid. The patient completed 25 fractions of radiotherapy on September 30, 2022.

Surveillance CT on September 13, 2023, showed lesions interpreted as recurrence along the right pelvic wall and in the left nephrectomy bed, together with dilatation of the residual left ureter. Palliative ablation of the left renal fossa lesion was considered in November 2023. During 2024, recurrent disease extended through the pelvis, retroperitoneum, and urinary tract. Abdominal wall, liver, lung, and nodal metastases followed, with worsening right hydroureteronephrosis (Figure 4 and Supplementary Figure S1). A later enhancing lesion was inseparable from the right ureter, with segmental narrowing and upstream dilatation. No surgical specimen was obtained; on imaging, however, the pattern was consistent with tumor-related ureteral involvement. Organ function deteriorated, and the patient died of multiorgan failure in June 2024, approximately 29 months after renal rupture. Supplementary Figure S2 summarizes the clinical course.

**Figure 4:**
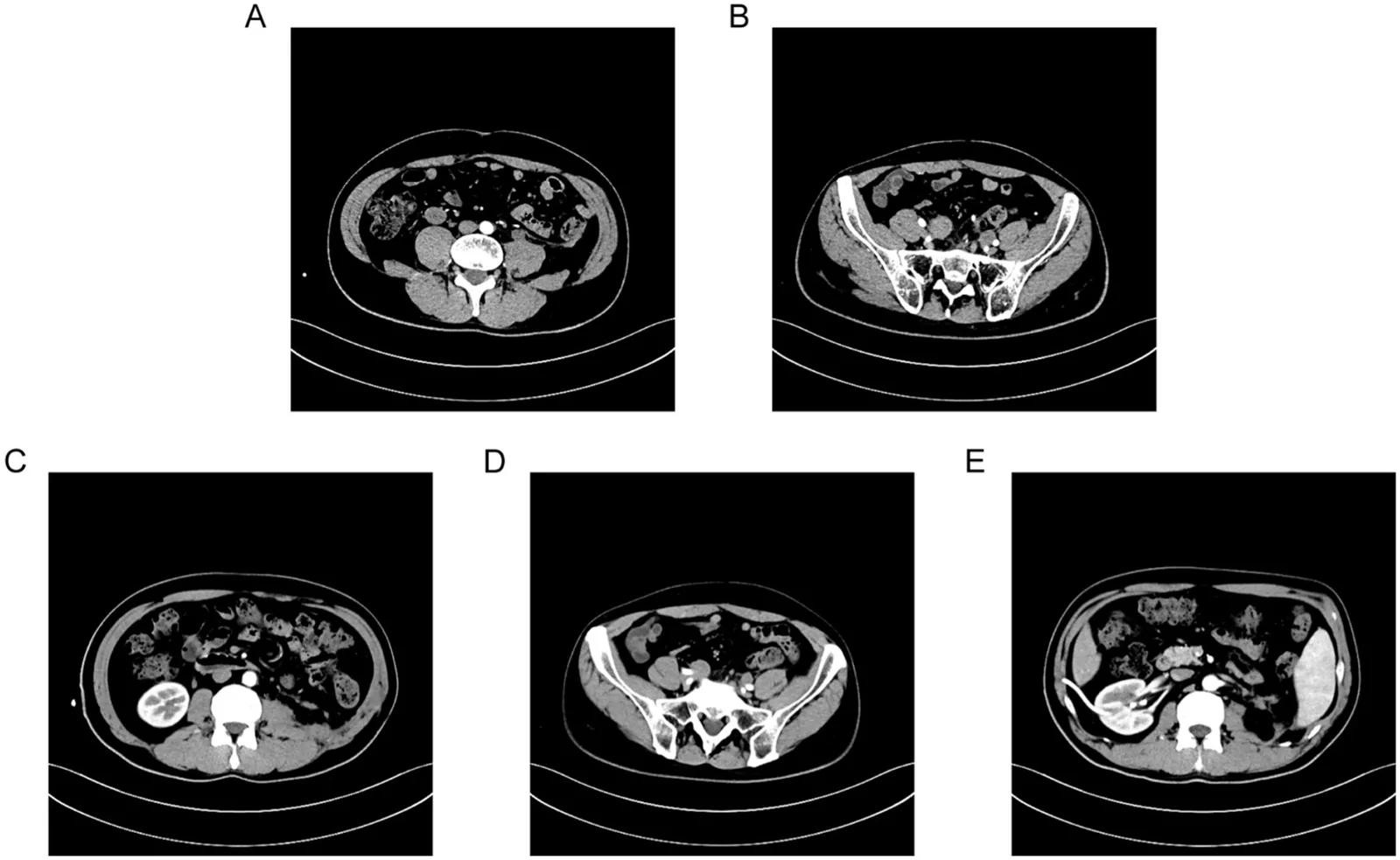
Follow-up changes in the upper urinary tract and retroperitoneum. **(A,B)** Follow-up CT showed abdominal and retroperitoneal recurrent lesions. **(C)** Right ureteral dilatation. **(D)** Dilatation of the residual left ureteral stump with concurrent right ureteral dilatation. **(E)** Changes after right upper urinary tract drainage.

## 3 Discussion

The main diagnostic difficulty was the absence of a visible mass. Contrast-enhanced CT, operative inspection, and gross examination showed no discrete renal or ureteral tumor, whereas histology revealed diffuse colorectal-type carcinoma with extensive necrosis and invasion of renal hilar vessel walls. Renal involvement first became clinically evident as rupture. At the same time, obstruction of the opposite upper urinary tract complicated emergency treatment.

No single mechanism fully explains the rupture. Longstanding left ureteral obstruction and repeated D-J stenting may have reduced the structural and functional reserve of the kidney, increasing its susceptibility to parenchymal disruption (17). Diffuse tumor infiltration and necrosis may also have weakened the renal architecture, and invasion of hilar vessel walls may have increased vascular fragility. The available sections do not show whether vessel invasion was located near the cortical tear. We therefore cannot assign it a direct causal role or use it as evidence of hematogenous spread. The oncology record documents eight cycles of XELOX ending on February 7, 2020. No bevacizumab or other vascular endothelial growth factor-directed treatment was recorded before the rupture. Because the patient was treated at several institutions, undocumented outside exposure cannot be excluded, but the available evidence does not support antiangiogenic therapy as a contributor.

The absence of a mass also separates this case from most previous reports. Wolff et al. described the closest precedent: spontaneous rupture of a renal metastasis from colon adenocarcinoma (13). In our patient, however, neither CT nor operative or gross examination disclosed a discrete mass. The opposite upper urinary tract was also obstructed, which influenced emergency planning. The other reports in Table 1 described papillary, isolated renal, or combined renal and ureteral lesions without spontaneous renal rupture (8–11, 14). In this case, diffuse tumor permeation and hemorrhage obscured the renal contour. In a patient with previous malignancy and otherwise unexplained SRR, the absence of an enhancing mass lowers suspicion but does not exclude malignant involvement. Diagnosis may depend on adequate tissue sampling and correlation with immunohistochemistry. Here, the immunophenotype supported colorectal origin but did not identify the route to the kidney. Later disease in the bladder, residual ureter, pelvis, and distant organs showed an aggressive course. It did not resolve whether spread was hematogenous, lymphatic, retrograde, or contiguous.

**Table 1:** Selected published reports of colorectal cancer involving the kidney or upper urinary tract compared with the present case.

Report	Year	Primary tumor	Renal/urinary tract involvement	Presentation and imaging	Key relevance/limitations
Wolff et al. (13)	1994	Colon adenocarcinoma	Renal metastasis	Spontaneous kidney rupture due to renal metastasis from colon adenocarcinoma	Previously reported CRC-related renal metastasis presenting with spontaneous renal rupture
Kibar et al. (14)	2005	Sigmoid adenocarcinoma	Unilateral renal papillary metastasis	Renal papillary localization without obvious metastatic disease in the publication abstract	Sigmoid cancer involvement of the renal papilla; SRR was not reported
Brambilla et al. (11)	2007	Colon cancer	Isolated renal metastasis	Reported as isolated renal metastasis after colon cancer	Renal recurrence after colon cancer; rupture was not reported
Dulskas et al. (9)	2015	Sigmoid adenocarcinoma	Renal metastasis	Rare case and literature review of sigmoid adenocarcinoma metastasizing to the kidney	Sigmoid adenocarcinoma involving the kidney; rupture was not reported
Huai et al. (8)	2022	Rectal adenocarcinoma	Renal and ureteral metastases	Renal and ureteral metastatic involvement	CRC involvement of the kidney and ureter; rupture was not reported
Saadaat et al. (10)	2025	Colon adenocarcinoma	Renal metastasis	Case report of renal metastasis from colon adenocarcinoma	Renal metastasis from colon adenocarcinoma; occult rupture was not reported
Present case	2022	Sigmoid adenocarcinoma with nodal involvement	Left renal involvement with SRR; chronic left-sided obstruction and concurrent moderate right hydroureteronephrosis	No discrete renal mass on CT, intraoperative inspection, or gross examination; diagnosis established by histopathology and IHC	Diffuse renal involvement without a discrete mass; contralateral D-J stenting preceded left nephrectomy

Search note: A focused PubMed search covered database inception through September 4, 2026, using combinations of ‘colorectal/colon/rectal cancer,’ ‘renal/kidney/ureteral metastasis,’ ’spontaneous renal rupture,’ and ‘Wunderlich syndrome.’ We reviewed English-language reports of colorectal adenocarcinoma involving the kidney or upper urinary tract. The search was non-systematic, and the table is illustrative rather than exhaustive.

CRC, colorectal cancer; D-J, double-J; IHC, immunohistochemistry; PET/CT, positron emission tomography/computed tomography; SRR, spontaneous renal rupture.

The pathological diagnosis was not yet available when the emergency treatment decision was made. Selective renal arterial embolization was considered to preserve the kidney. Open nephrectomy was chosen because bleeding appeared to be continuing, the chronically obstructed left kidney was thought to have little salvageable function, and malignant rupture could not be excluded. The right kidney was also obstructed and would become the only renal unit, so a right D-J stent was placed first. This sequence was specific to the circumstances of this patient and should not be taken to mean that nephrectomy is generally preferable to embolization in SRR. Decisions in similar cases should take account of hemodynamic status, the likely source of bleeding, ipsilateral renal reserve, and drainage of the contralateral kidney.

Some records could not be recovered because the patient received care at several institutions. Contemporaneous documents from the principal treating hospitals still allowed us to reconstruct the emergency sequence, including serial hemoglobin measurements, transfusion, and operative blood loss. By the time of surgery, a large hematoma had already formed. The transfusion requirement therefore reflects the entire hemorrhagic episode, not only the estimated operative loss of 400 mL. These documentary gaps do not change the histopathological diagnosis of diffuse colorectal-type carcinoma involving the kidney.

The 2019 stage remains difficult to interpret. The source report assigned pT4bN1bM1a, so renal involvement found in 2022 may represent progression of disease already considered metastatic in 2019 rather than new dissemination. However, the anatomical basis for M1a was not recorded, and earlier imaging showed no renal mass. The stage designation alone cannot establish when or where renal involvement began. Only 10 lymph nodes were assessed, below the conventional 12-node benchmark. The available records confirm XELOX through February 2020, surveillance CT in January 2021, and left ureteral stenting in March 2021, with no evidence of bevacizumab or another antiangiogenic agent before rupture. Although the April 2022 right ureteral biopsy was non-diagnostic, later CT showed an enhancing lesion inseparable from the ureter, with narrowing and upstream dilatation. The imaging supports tumor-related ureteral involvement, but without later surgical tissue, mural invasion cannot be confirmed histologically. The evidence is therefore stronger for colorectal origin and progressive urinary tract disease than for any particular route of spread.

An absent mass does not rule out malignant renal involvement in a patient with previous colorectal cancer and otherwise unexplained nontraumatic renal or perirenal hemorrhage. Adequate histological sampling may reveal diffuse disease missed by imaging and gross examination. If nephrectomy is required and the contralateral upper urinary tract is obstructed, drainage of the remaining renal unit should be planned before surgery.

## Patient perspective

A patient perspective was not obtained before the patient’s death and is therefore not available for this report.

## Data Availability

The original contributions presented in the study are included in the article/Supplementary Material, further inquiries can be directed to the corresponding author/s.

## Supplementary material

The Supplementary Material for this article can be found online at: https://www.frontiersin.org/articles/10.3389/fsurg.2026.1954420/full#supplementary-material

SUPLLEMENTARY FIGURE S1 Advanced urinary tract and retroperitoneal progression. Axial contrast-enhanced CT images are arranged from superior to inferior. (A) Recurrent soft tissue in the left nephrectomy bed involved the residual left ureteral stump (red arrow); persistent right ureteral dilatation was also visible (blue arrow). (B) At a more caudal retroperitoneal level, an enhancing right retroperitoneal soft-tissue lesion (red arrow) was contiguous with the proximal right ureter. (C) Further inferiorly, focal enhancing soft tissue (red arrow) remained inseparable from the right ureter, with segmental narrowing and upstream ureteral dilatation. (D) An intravesical lesion (red star) was visible on follow-up CT.

SUPLLEMENTARY FIGURE S2 Clinical timeline and sequence of the January 2022 urological emergency. The upper row summarizes the course from sigmoid colectomy in 2019 to death in June 2024. The lower panel shows the emergency sequence. At presentation, hemoglobin was 108 g/L and creatinine 329 μmol/L. The right collecting system was drained before open left nephrectomy. Estimated operative blood loss was 400 mL; 6 U of red blood cells and 600 mL of plasma were transfused during the hemorrhagic episode. Hemoglobin was 94 g/L later that day and 83 g/L the next morning. Histology showed diffuse colorectal carcinoma involving the kidney despite the absence of a discrete renal mass. Cr, creatinine; D-J, double-J; Hb, hemoglobin; RBC, red blood cells.
