# Supplementary material for: Occult renal involvement by sigmoid colon adenocarcinoma presenting as spontaneous renal rupture: a case report
Source: Front Surg. 2026 Oct 30;13:1954420. doi: 10.3389/fsurg.2026.1954420 (PMC13759826; doi:10.3389/fsurg.2026.1954420)

Clinical timeline and January 2022 emergency sequence

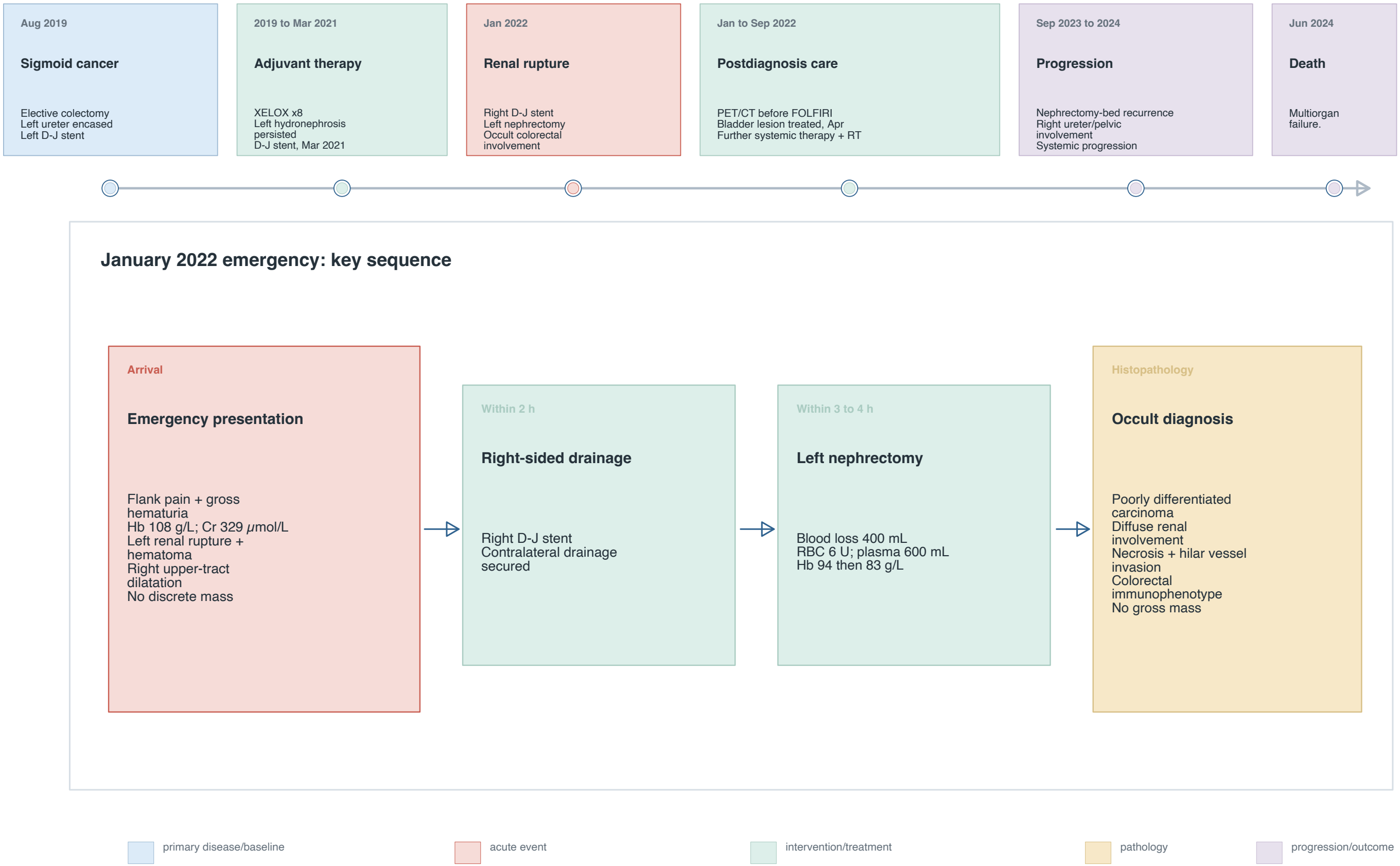

Supplement: Supplementary file 2 [file Datasheet2.pdf]
